# Leptospirosis in Vellore: A Clinical and Serological Study

**DOI:** 10.1155/2014/643940

**Published:** 2014-06-23

**Authors:** G. Vimala, A. Mary Josephine Rani, V. Raja Gopal

**Affiliations:** ^1^Department of Zoology, Auxilium College, Vellore, Tamil Nadu 632006, India; ^2^Zonal Entomological Team, Vellore, Tamil Nadu 632001, India

## Abstract

Leptospirosis is a severe spirochetal zoonosis in the world. It is considered an occupational disease of persons engaged in agriculture, sewage works, forestry, and animal slaughtering. A study was conducted with an objective of assessing the seroprevalence of leptospirosis in and around Vellore district, Tamil Nadu. The study was based on the signs and symptoms of the patients reporting fever in Vellore Municipal Clinic (Urban Malarial Scheme). Blood samples were collected from 129 patients. Animal studies were conducted from 24 rodents captured form the market place of the Vellore municipality. In the ZET (Zonal Entomological Team, Vellore) Laboratory the sera were examined by macroscopic slide agglutination test (MSAT). In the MAST, totally 10 positive leptospiral cases from human beings and 10 positive leptospiral cases from rats (*Rattus rattus* and *Rattus norvegicus*) were found out. Then both positive cases of leptospiral vials were labeled, sealed, and sent to the Leptospirosis Research Laboratory, Madhavaram, Chennai, for further serovars examination. Among the various serovars identified *autumnalis* was more prevalent. Our findings showed that the age groups between 15 and 55 years showed more susceptibility. Particularly the adults were more infected. The majority of seropositive individuals in the cases had only subclinical infection. Rodents were abundant and contributed to enzootic and endemic prevalence of leptospirosis.

## 1. Introduction

Leptospirosis is a common zoonosis worldwide that affects mammals, including human beings. Infection is endemic and occurs with greatest frequency in tropical and subtropical regions. It is emerging as an important public health problem in India [1–3]. Both humans and animals can be directly infected through contact with infected tissue or urine or indirectly through contact with contaminated soil and water [4, 5]. In humans, typical symptoms can include fever, headaches, chills, vomiting, sore muscles, jaundice, red eyes, abdominal pain, diarrhea, and rashes. Leptospirosis can become considerably dangerous if not treated, potentially leading to kidney damage, meningitis, liver failure, and respiratory problems [5].

Leptospirosis has been recognized as an important occupational hazard of agriculture manual laborers, sewage workers, animal handlers, forestry workers and other outdoor workers who work in wet conditions, and butchers. The transmission cycle involves interaction between one or more animal hosts harboring leptospires, an environment favorable for its survival, and human beings. Leptospirosis in human occurs in two courses: anicteric or benign (between 85 and 90% of cases) and icteric or serious, also known as Weil's diseases (between 10 and 15% of cases). The wide spectrums of clinical symptoms that characterize leptospirosis make its diagnosis easily confused with other febrile diseases [6].

*Leptospira interrogans,* which causes human leptospirosis, consists of over 24 serogroups made up of a large and expanding number of serovars. Serogroups and their member serovars causing leptospirosis differ from region to region. These are identified by specific laboratory tests like isolation of organism by culture, specific antigen detection by gene amplification by polymerase chain reaction (PCR), or antibody detection by microscopic agglutination test (MAT). MAT uses whole leptospire as antigen and detects both lgM and lgG antibodies. IgM antibodies may disappear after six months and IgG antibodies persist for two to 10 years after infection [7]. MAT has a high degree of specificity, identifies serogroups, and is accepted worldwide, but it is expensive, time consuming, and labour intensive.

Knowledge of high level of exposure and prevalence of leptospirosis in an area helps the primary physician in deciding to start early antibiotic treatment on clinical assessment. Antibiotic treatment should be started as soon as possible on clinical suspicion of leptospirosis because organ damage sets in by the second half of first week itself and late antibiotic treatment does not influence the outcome [8] (some strains like* lai* may cause earlier mortality in the first three days [9]). Presently available culture and antibody based tests become positive only after the end of first week of infection and are unhelpful in deciding to start antibiotics early.

Developing vaccines for domestic animals and humans to bring down the number of cases in endemic areas is important. China, Japan, and Cuba have reported the effectiveness of regionally made vaccines [10]. Vaccines should be specific for the group of strains particular to a region; hence the identification of local serogroup(s) is important [11]. Documenting high level of seroprevalence among rodents is also important to impress upon the general public the need for rodent control and protective barriers for farmers as important methods of control of leptospirosis.

Leptospirosis appears to be on the increase in Kerala, Tamil Nadu, and Andamans during the last two decades, probably due to increased farming and inadequate rodent control [12]. Since the 1980s, outbreaks are being increasingly reported especially from the states of Tamil Nadu, Kerala, and Karnataka [13]. Till May 2001, 16 health units including Chennai have recorded cases of leptospirosis. A total of 617 cases have been reported. Major contribution to the number of cases had been rendered by Chennai with 509 cases. Other units reporting cases are Kanchipuram, Cuddalore, Pudukkottai, Thiruvannamalai, Tirupattur, Kanyakumari, Tiruppur, Vellore, Villupuram, and Erode [14].

In response to this need, the present study is an integrated approach including analysis of seroprevalence, incidence, most common clinical course, circulating serovars, and transmission factors of leptospirosis in and around Vellore zone.

## 2. Materials and Methods

### 2.1. Study Area

The town of Vellore, Tamil Nadu, is situated on the west of the Palamathi Hill range. The river Palar flows on the north of the town. The town is located between 12.0°–13.35° North and 78.3°–80.2° East. The area is characterized by a tropical climate. According to the meteorological data acquired the meteorological observatory, the area Vellore had a high mean relative humidity (75%) except from March to June. The mean monthly temperature ranges from a low of 16.9°C in December to a high of 40°C in May. The area receives rainfall from southwest monsoon.

### 2.2. Study Design

The study design includes the detection of different leptospiral serovars from patients reporting fever and rodents; particularly rats are chosen for the study because they are the source of most human cases of leptospirosis. The study was conducted from October 2009 and extended up to March 2010. The office of Senior Entomologist of Zonal Entomological Team was contacted and proper permission to study the rate of prevalence among patients has been obtained. The study was based on the signs and symptoms of the patients reporting fever in Vellore Municipal Clinic (Urban Malarial Scheme).

### 2.3. Serum Collection from Human Beings

The blood was taken from 129 patients having signs and symptoms of leptospirosis such as fever, headache, chills, rashes, body pain, vomiting, cough, abdominal pain, jaundice, and myalgia. 3 mL of intravenous blood was collected from patients and coagulated by centrifuging at 1500 rpm for 15 minutes. The resulting serum was stored in Eppendorf tubes at −20°C until use. Details of sera sample number, patient name, age, sex, date of collection, address, duration of illness, and symptoms of the illness were also recorded.

In the ZET (Zonal Entomological Team, Vellore) Laboratory the sera were examined by Anti-*Leptospira* IgM Dipsticks* Leptospira* test (macroscopic slide agglutination test (MSAT)) which was used to detect positive cases. Then positive cases of leptospiral vials were labeled, sealed, and sent to the Leptospirosis Research Laboratory, Madhavaram, Chennai, for further serovars' examination. In the laboratory the sera were examined by microscopic agglutination test (MAT). MAT was valuable in identifying the serovars. The antigen panel included the following 12 serovars:* icterohaemorrhagiae, canicula, grippotyphosa, hebdomadis, pomona, autumnalis, pyrogenes, tarassovi, ballum, australis, javanica,* and* hardjo*. The cutoff point was dilutions ≥ 1 : 100 and the predominant serovar was that with the highest dilution.

Positive cases were analyzed using epidemiological data such as age, sex, regional distribution, and serovars as well as clinical data like signs and symptoms. All data were recorded and analyzed.

### 2.4. Serum Collection from Rodents

About 24 rats were captured live from the market place of the Vellore Municipality, the Mundy Street. Then the speciation of the rats was carried out. The rats were identified by studying their morphology. Five rats belong to the species* Rattus rattus* (house rat) and another 19 rats belong to the species* Rattus norvegicus* (godown rat). Then the rats were anaesthetized and dissected. Sufficient blood was collected through piercing of the heart. Serum was separated by centrifugation and stored at −20°C until use. Anti-*Leptospira* IgM Dipsticks* Leptospira* test (macroscopic slide agglutination test (MSAT)) was used to detect positive cases and MAT (microscopic agglutination test) was used to determine serovars. Antigen and cut-off values were as those used for the human seroprevalence study.

## 3. Results

Serum samples were collected during the period of October 2009, November 2009, and February 2010 from a total of 129 febrile patients and 24 rats having signs and symptoms of leptospirosis. From the collected serum samples tested by means of macroscopic slide agglutination test (MSAT), 10 out of 129 patients and 10 out of 24 rats were found positives (Table 1). In human beings out of 10 positive cases, 5 cases were found to be males and 5 cases were found to be females.

Table 2 represents the agewise distribution of leptospiral antibodies in humans. Below the age of 15 one case was found to be positive for leptospirosis, between the ages of 16 and 25 two cases were found to be positive for leptospirosis, between the ages of 26 and 35 two cases were found to be positive for leptospirosis, between the ages of 36 and 45 two cases were found to be positive for leptospirosis, and between the ages of 46 and 55 one case was found to be positive for leptospirosis, followed by two cases above the age of 55 which were found to be positive for leptospirosis.

Table 3 showed the clinical profile among 10 positive cases. Fever was reported by all positive cases. Fever and headache were reported by 7 cases, fever, headache, and myalgia by 6 cases, fever, headache, myalgia, and body pain by 4 cases, and fever, myalgia, body pain, and vomiting by 1 case.

Table 4 represents the fever duration among the positive cases. Fever was found to have lasted for less than 5 days among one positive case and for more than 5 days and less than 10 days among 5 positive cases. Among positives one case had fever for more than 10 days and less than 15 days and for more than 20 days and less than 25 days in one positive case. Fever lasts more than 1 month and 3 months were also reported.

Table 5 showed the clinical symptoms among 129 febrile cases. Fever was reported by all cases. Headache was reported by 107 cases, vomiting by 17 cases, and body pain by 71 cases. Cough was reported by 13 cases, myalgia by 56 cases, and abdominal pain by 10 cases. Other symptoms like nausea and anorexia were reported by 8 cases.

There are 12 leptospiral serovars (*canicula, icterohaemorrhagiae, grippotyphosa, hebdomadis, pomona, autumnalis, pyrogenes, tarassovi, ballum, javanica, australis, *and* hardjo*) which were tested in human beings and rats. Out of 10 positives human being cases, the serovar* autumnalis* was found to be present only in one case. 10 positive rat samples were tested for serovars identification. None were identified from the positive cases.

## 4. Discussion

The main aim of the study was to validate the case definition. In this study the survey conducted during the monsoon season, that is, October and November, and after the monsoon season, that is, February, shows an increase in the prevalence of leptospirosis. The highest number of cases was reported in the months of November and February. These findings correlate with the findings of the study by Ko et al. [15], which showed that leptospirosis could be the cause of many febrile illnesses in urban slums during and after the monsoon seasons. According to the meteorological data those months were characterized by rainfall. This suggests that rain does play an important role in the dissemination of the disease to human beings. Epidemics associated with high case fatality (greater than 15%) break out annually during seasonal periods of heavy rainfall in poor urban areas that lack basic sanitation infrastructure [16]. Rainfall and exposure were defined as an important correlation in diagnosing patients with leptospirosis in India [17]. Since Vellore experienced scanty rainfall this monsoon, it would have resulted in the minimal dissemination of the disease in human beings with 8.1% during the monsoon and post monsoon season. If the rainfall had been heavy, the percentage would have been more. Since rainfall plays an important part in the dissemination of disease, the basic sanitation infrastructure needs to be improved in the Vellore urban zone.

The study proves the point that leptospirosis mostly occurs in working age groups and also in children. The disease was more prevalent in the age groups of 15 to 55. These findings contradict with the study by Davol [18] which showed the mean age of the confirmed cases of leptospirosis was 39 years. Increasing age and multiple chronic health conditions can increase the severity of these syndromes and the potential for death [19]. Also, children can be at risk by playing in infected water or having contact with infected animals [20]. After the flood occurred in Mumbai in July 2000, a total of 53 children below the age of 12 years were admitted to a pediatrics department and were tested for leptospirosis. Serological results indicated that almost one-third of the children had acute leptospirosis [21]. However, the highest mortality rates remain among the elderly and those with Weil's syndrome [22].

The study has also confirmed the male to female ratio was 1 : 1. Leptospirosis is more prevalent in men and also in women; that is, both are equally affected because of the fact that nowadays both of them were more exposed to the contaminated environment. This finding contradicts the findings of the study by Davol [18] which showed the male to female ratio was 3 : 1. That is leptospirosis was more prevalent in men than women.

All patients included in the study had fever. The commonest symptoms of those who were confirmed as having leptospirosis were fever, headache, myalgia, and vomiting; other symptoms were not that common. These findings correlate with the findings of the study by Davol [18] which showed all positive cases having symptoms like fever, headache, and myalgia. In this study, the leptospirosis was more prevalent in the cases having fever more than 5 days and less than 10 days among 5 positive cases. Fever which was found to have lasted for less than 5 days in one positive case was also reported. Fever which lasts more than 1 month and 3 months was also reported. These findings correlate with the findings of the study by Rao et al. [23] which showed the duration of fever of leptospirosis mostly occurs with a range of 2–20 days and lasts for a month also. These findings also correlate with the findings of the study by Chrispal et al. [24] which showed that a total of 398 acute undifferentiated febrile illnesses (duration 5–21 days) of patients were diagnosed in tertiary care hospital in South India proven scrub typhus (47.5%); malaria (17.1%); enteric fever (8.0%); dengue (7.0%); leptospirosis (3.0%); spotted fever rickettsiosis (1.8%); hantavirus (0.3%); and others. Most individuals recover from leptospirosis within 6–12 weeks after the onset of illness. The symptoms like fever, headache, and vomiting are most noticeable during the 4–17 days following infection; then the fever normally decreases and bacterial lysis occurs [23].

Totally 233 batteries of* Leptospira* antigens (serovars) were present. In Leptospirosis Research Laboratory, Madhavaram, Chennai, only 12 batteries of* Leptospira* antigens (*canicula, icterohaemorrhagiae, grippotyphosa, hebdomadis, pomona, autumnalis, pyrogenes, tarassovi, ballum, javanica, australis, *and* hardjo*) were used to find out the serovars. Out of 10 positives human being cases, the serovar* autumnalis* was found to be present in only one case. The positive serovar case was female above 55 years and the duration of fever was 6 days. Thus remaining serovars may be present in other positive cases. In this study white fancy rats domestic albino (*Rattus norvegicus*) and house rats (*Rattus rattus*) were identified as the potential infection source for acute leptospirosis in humans. 10 positive rat samples were tested for serovars identification. No serovars were present among the positive cases because only 12 batteries of* Leptospira* antigens were used. Thus remaining serovars may be present. Some signs and symptoms are more common with particular serovars. For example, jaundice occurs in 83% of individuals with serovar* icterohaemorrhagiae* infection and in 30% of individuals with serovar* pomona* infection. Serovar* autumnalis* infections produce a unique pretibial erythematous rash [19].

Foregoing facts denote that the epidemiology of leptospirosis is complex. In a given area one animal species harbours one serotype or, at most, a few serovars that are weakly differentiated antigenically. It is also obvious that epidemiology of leptospirosis is dynamic and ever changing, thus responding to the ecological changes. The public health importance of leptospirosis lies in its occupational, seasonal, sex, and age related incidence.

A further problem that cannot be ignored is the fact that most people at risk of leptospirosis live in developing countries without ready access to specialized diagnostic or treatment facilities. Knowledge and research in leptospirosis are not available in most developed countries to which developing countries turn for scientific assistance. The situation is aggravated when inadequate statistics in developed countries indicate a spuriously low rate of leptospirosis, leading to an impression that the disease is not existent or unimportant and does not justify allocation of funds for the maintenance of existing research surveillance or for new studies toward solutions to the problems. The purpose of this experimental study is to present analytical data so as to provide information regarding the prevalence of leptospirosis in Vellore zone. Due to the derth of manpower and time, this experimental study could be conducted and details are gathered from a limited number of samples only. Serological test in leptospirosis will help the clinicians to confirm the diagnosis and start the treatment at an early stage. Thus, these findings may help devise a better control strategy against the disease in this zone.

## Figures and Tables

**Table 1:** Prevalence of leptospiral antibodies among patients and rats screened.

S. number	Type of serum samples	Number of samples tested	Number of positives	Percentages
1	Human beings	129	10	7.75%
2	Rats	24	10	41.66%

**Table 2:** Agewise distribution of patients positive for leptospiral antibodies.

S. number	Age	Positive cases	Percentages
1	Below 15	1	10%
2	16–25	2	20%
3	26–35	2	20%
4	36–45	2	20%
5	46–55	1	10%
6	Above 55	2	20%

**Table 3:** Clinical symptoms among patients positive for leptospiral antibodies.

S. number	Clinical symptoms	Number of cases	Percentages
1	Fever	10	100%
2	Fever + headache	7	70%
3	Fever + headache + myalgia	6	60%
4	Fever + headache + myalgia + body pain	4	40%
5	Fever + myalgia + vomiting + body pain	1	10%

**Table 4:** Duration of leptospiral fever among the positive cases.

S. number	Number of days of fever	Male	Female	Total
1	Below 5 days	—	1	1
2	5–10 days	3	2	5
3	10–15 days	1	—	1
4	15–20 days	—	—	—
5	20–25 days	1	—	1
6	1 month	—	1	1
7	3 months	—	1	1

**Table 5:** Clinical symptoms of all the patients screened for leptospirosis.

S. number	Clinical symptoms	Number of cases	Percentages
1	Fever	129	100%
2	Head ache	107	82.9%
3	Vomiting	17	13.2%
4	Body pain	71	55%
5	Cough	13	10%
6	Myalgia	56	43.4%
7	Abdominal pain	10	8.2%
8	Others (nausea, anorexia)	8	6.2%
